# Outbreak of *Fusarium solani* Meningitis in Immunocompetent Persons Associated With Neuraxial Blockade in Durango, Mexico, 2022–2023

**DOI:** 10.1093/ofid/ofad690

**Published:** 2024-01-04

**Authors:** Gabriel García-Rodríguez, Célida Duque-Molina, Irasema Kondo-Padilla, Christian Arturo Zaragoza-Jiménez, Vladimir Brian González-Cortés, Rocio Flores-Antonio, Tania Villa-Reyes, Adriana Vargas-Rubalcava, Luis Ángel Ruano-Calderon, Juan Carlos Tinoco-Favila, Héctor Carlos Sánchez-Salazar, Rodolfo Rivas-Ruiz, Octavio Castro-Escamilla, Rosa Areli Martínez-Gamboa, Fernanda González-Lara, Irma López-Martínez, Tom M Chiller, Rosana Pelayo, Laura C Bonifaz, Zoe Robledo-Aburto, Jorge Alcocer-Varela

**Affiliations:** Dirección General de Epidemiología, Secretaría de Salud, Mexico City, Mexico; Dirección de Prestaciones Médicas, Instituto Mexicano del Seguro Social, Mexico City, Mexico; Dirección de los Servicios de Salud del Estado de Durango, Secretaría de Salud, Durango, Mexico; Dirección de Información Epidemiológica, Dirección General de Epidemiología, Secretaría de Salud, Mexico City, Mexico; Coordinación de la Red Hospitalaria de Vigilancia Epidemiológica, Dirección de Información Epidemiológica, Secretaría de Salud, Mexico City, Mexico; Coordinación de la Red Hospitalaria de Vigilancia Epidemiológica, Dirección de Información Epidemiológica, Secretaría de Salud, Mexico City, Mexico; Coordinación de la Red Hospitalaria de Vigilancia Epidemiológica, Dirección de Información Epidemiológica, Secretaría de Salud, Mexico City, Mexico; Coordinación de la Red Hospitalaria de Vigilancia Epidemiológica, Dirección de Información Epidemiológica, Secretaría de Salud, Mexico City, Mexico; Servicio de Neurología, Hospital General 450, Servicios de Salud del Estado de Durango, Secretaría de Salud, Durango, Mexico; Clínica de Infectología, Hospital General 450, Servicios de Salud del Estado de Durango, Secretaría de Salud, Durango, Mexico; Unidad de Cuidados Intensivos, Hospital General de Zona 1, Instituto Mexicano del Seguro Social, Durango, Mexico; División de Investigación Clínica, Coordinación de Investigación en Salud, Instituto Mexicano del Seguro Social, Mexico City, Mexico; División de Investigación Clínica, Coordinación de Investigación en Salud, Instituto Mexicano del Seguro Social, Mexico City, Mexico; Laboratorio de Microbiología Clínica, Servicio de Infectología, Instituto Nacional de Ciencias Médicas y Nutrición Salvador Zubirán, Mexico City, Mexico; Laboratorio de Microbiología Clínica, Servicio de Infectología, Instituto Nacional de Ciencias Médicas y Nutrición Salvador Zubirán, Mexico City, Mexico; Dirección de Diagnóstico y Referencia, Instituto de Diagnóstico y Referencia Epidemiológicos, Secretaría de Salud, Mexico City, Mexico; Mycotic Diseases Branch, Centers for Disease Control and Prevention, Atlanta, Georgia, USA; Unidad de Educación e Investigación, Dirección de Prestaciones Médicas, Instituto Mexicano del Seguro Social, Mexico City, Mexico; Coordinación de Investigación en Salud, Dirección de Prestaciones Médicas, Instituto Mexicano del Seguro Social, Mexico City, Mexico; Dirección General, Instituto Mexicano del Seguro Social, Mexico City, Mexico; Dirección General, Secretaría de Salud, Mexico City, Mexico

## Abstract

**Background:**

Fungal meningitis can be associated with epidural anesthesia procedures. Fusariosis is a rare infection typically affecting immunocompromised patients and rarely causes meningitis. During 2022–2023, public health officials responded to a large outbreak of *Fusarium solani* meningitis associated with epidural anesthesia in Durango, Mexico.

**Methods:**

The public health response and epidemiological and clinical features of patients affected by this outbreak were described. Coordinated actions were addressed to identify the etiological agent, determine its drug susceptibility, develop diagnostic tests, and implement clinical and epidemiological protocols. Retrospective analyses of clinical variables and outcomes were performed to determine association with better patient survival.

**Results:**

A total of 1801 persons exposed to epidural anesthesia were identified, of whom 80 developed meningitis. *Fusarium solani* was found in 3 brain biopsies and showed susceptibility to voriconazole and amphotericin B. After *F solani* polymerase chain reaction (PCR) implementation, 57 patients with meningitis were PCR-screened, and 31 (38.8%) had a positive result. Most patients were female (95%), and cesarean section was the most common surgical procedure (76.3%). The case fatality rate was 51.3% (41 patients) and the median hospitalization duration was 39.5 days (interquartile range, 18–86 days). Seventy-one patients (88.8%) received voriconazole/amphotericin B and 64 subjects (80%) additionally received steroids. Cox regression analysis showed an increased lethality risk in patients who received antifungal treatment after 5 days (hazard ratio, 2.1 [95% confidence interval, 1.01–4.48], *P* < .05).

**Conclusions:**

The *F solani* meningitis outbreak in Durango was an unprecedented medical challenge. Timely treatment and effective healthcare management were associated with better survival outcomes.

## Teaser

During 2022–2023, an outbreak *of Fusarium solani*–related meningitis occurred in Durango, Mexico, affecting 80 patients with a mortality of 51.3%. Prompt antifungal treatment and increased health awareness were associated with patient survival.

*Fusarium solani* belongs to a family of phytopathogenic filamentous fungi that are widely distributed in the environment [1, 2]. Immunocompromised patients, such as those with hematological or solid malignancies or those receiving immunosuppressive therapies, may rarely develop invasive fusariosis; in rare instances, the fungus can disseminate to the central nervous system (CNS), producing meningitis [3–5]. Invasive fungal infections in immunocompetent persons are uncommon. In 2012, the Centers for Disease Control and Prevention (CDC) reported the first outbreak of fungal meningitis (primarily due to *Exserohilum rostratum*) in the United States (US) caused by the administration of contaminated methylprednisolone through epidural, paraspinal, or joint injections, with mortality rates close to 8% [6]. In May 2023, the CDC reported a Mexico–US binational outbreak of *F solani* meningitis associated with neuraxial blockade received during surgical procedures in Matamoros, Mexico [7]. Recent results from this outbreak showed a mortality rate close to 80%, patients developed cerebrovascular complications, and the isolated strain was resistant to conventional antifungal drugs such as voriconazole and amphotericin B (AmB) [8]. Here, we present data on the epidemiology, clinical features, and patient outcomes from the largest reported outbreak among immunocompetent persons of *F solani* meningitis associated with procedures performed under epidural anesthesia in Durango, Mexico, from May 2022 to February 2023.

## METHODS

### Index Case and Public Health Response

On 13 September 2022, the Regional Hospital No. 1 of the Mexican Institute of Social Security (IMSS) in Durango, Mexico, received a female patient in the emergency room with persistent headache, photophobia, aphasia, and disorientation. The patient had no history of hematological or solid malignancies or bacterial infections; she had undergone a cesarean section (c-section) 2 months earlier under epidural anesthesia. During hospitalization, the patient continued with persistent severe headaches with no improvement to ceftriaxone, vancomycin, acyclovir, or antituberculosis drugs. On 5 October, the cerebrospinal fluid (CSF) sample showed hypoglycorrhachia, hyperproteinorrachia, and pleocytosis, and the lack of response to antibiotics, antiviral, and antituberculosis treatment continued. Eight days later, the patient was admitted to the intensive care unit (ICU) due to subarachnoid hemorrhage, developed transient cerebral ischemia and severe hemorrhage, and died on 24 November.

Fifteen days after the hospitalization of the first patient, 2 other female cases with similar symptoms were admitted to the Ministry of Health General Hospital of Durango. Both patients had undergone a c-section within 2 months before their hospitalization. On 3 November, the General Epidemiology Office was notified of cases of atypical meningitis in Durango, in patients with similar symptoms who underwent a c-section with epidural anesthesia at 4 private hospitals. Health and regulatory agencies proceeded to close these sites and implemented an active search to identify and contact all patients who received neuraxial blockade from May 2022 to date.

In response to this outbreak, the Public Health Incident Management Command took measures in epidemiological surveillance, medical care, public health policies, and health regulation, to address the challenges of atypical meningitis. On 11 November, the identification of the etiological agent was carried out in the Clinical Microbiology Laboratory at the National Institute of Medical Sciences and Nutrition Salvador Zubirán (INCMNZS), while the National Institute of Epidemiological Reference and Diagnosis (InDRE) standardized the CSF real-time PCR (qPCR) technique for *F solani*. By November 22, the Public Health Incident Management Command implemented the first protocol for fungal meningitis (“Protocol of care for cases of fungal infections of the central nervous system associated with neuraxial blockade”), indicating patient management in both the hospital and ambulatory settings, which was updated and published on 6 December [9].

### Study Design and Population

This is a retrospective cohort of 80 meningitis cases (Table 1) who were exposed to surgical procedures with neuraxial blockade in private hospitals in the state of Durango, Mexico, and developed symptoms and neurological alterations from May 2022 to February 2023 (when the last case was reported) [10]. Overall, 80 patients were admitted to public hospitals in Durango, Mexico, and were followed up until hospital discharge or death. Data on epidemiological, demographic, and clinical characteristics (surgical procedure, PCR result, days of hospitalization, days from surgery to hospitalization, ICU admission, percentage of patients with antifungal treatment and onset time, and steroid administration) were registered on a standardized case report form by the Ministry of Health, General Epidemiology Office, IMSS, and the Institute for Social Security and Services for State Workers.

**Table 1.** Case Definitions for the Durango Outbreak

Term	Definition
Exposed	All persons exposed to epidural anesthesia from May 2022 to February 2023 at any of the 4 private hospitals under investigation in Durango, Mexico
Suspected	All subjects exposed with symptoms suggestive of CNS infection (headache, neck pain, seizures, mental alterations)
Probable	Subjects exposed or suspected with 1 or more CSF alteration (eg, hypoglycorrhachia, hyperproteinorrachia, or pleocytosis)
Meningitis case	All classified subjects according to any of the previous definitions and with the following criteria: (1) Association with clinical-epidemiological data: (a) Patients without a *Fusarium solani* confirmatory test (culture or molecular), whose symptoms of meningitis or CSF alterations were not explained by another cause (2) Confirmation by laboratory test: (b) *Fusarium solani* culture from CSF or brain biopsy (c) Subjects with positive qPCR test for *F solani*

Abbreviations: CNS, central nervous system; CSF, cerebrospinal fluid; qPCR, real-time polymerase chain reaction.

### Microbiology Testing

Three samples from 2 patients (2 brain biopsies and 1 CSF specimen) were received and processed at the Clinical Microbiology Laboratory of the INCMNZS. Identification of the *F solani* sp complex was performed using 2 methods: mass spectrometry (matrix-assisted laser desorption/ionization–time of flight Bruker MS) and Sanger sequencing of the elongation factor 1α (EF1α) gene. After identification, InDRE standardized DNA extraction from CSF specimens through QIAamp DNA Mini Blood (Qiagen). The real-time PCR was developed and standardized using the Fusacept Kit (genes2life) for *F solani* detection. On 28 November 2022, qPCR was implemented at the Durango State Public Health Laboratory.

### Survival and Statistical Analysis

Categorical and continuous data were compared using Pearson χ^2^ and Mann-Whitney *U* tests, respectively. A receiver operating characteristic (ROC) was used to obtain the area under the curve (AUC), sensitivity, specificity, and Youden index to calculate the therapeutic time window of voriconazole/AmB to predict mortality, considering the elapsed time since hospital admission until the treatment initiation. The hazard ratio (HR) was determined with an unadjusted Cox regression model for late initiation of antifungal treatment and use of steroids. All statistical analyses were performed using SPSS software (IBM SPSS, version 26.0), and reported *P* values were 2-tailed, where a *P* < .05 value was considered significant.

## RESULTS

### Outbreak Description

**Figure 1.**
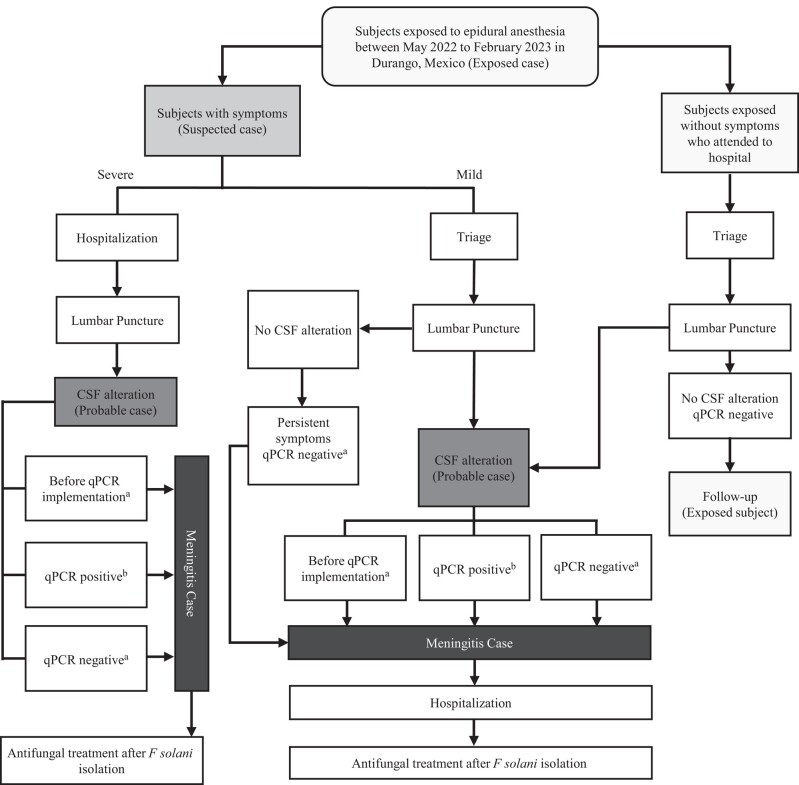
Algorithm for the active search of cases during the Durango *Fusarium solani* outbreak. An overall of 1801 persons were exposed to surgical procedures under neuraxial blockade in 4 private clinics between May 2022 to February 2023 in Durango, Mexico. The search of cases was carried out according to the presence of symptoms and cerebrospinal fluid (CSF) alterations. Subjects with severe or mild symptoms were classified as suspected cases and were immediately hospitalized or examined in the triage area, respectively. Lumbar punctures were performed and the presence of CSF alterations allowed the identification of probable cases, whereas the meningitis cases were identified by clinical-epidemiological data or by laboratory test (culture or real-time polymerase chain reaction [qPCR]). Some patients with mild symptoms and no CSF alterations were classified as meningitis cases based on their clinical-epidemiological data. Three exposed subjects without symptomatology showed CSF alterations whereas the rest of the exposed subjects were followed up in an ambulatory setting. The meningitis cases received antifungal drugs after *F solani* isolation. ^a^Subjects classified as meningitis cases by clinical-epidemiological association. ^b^Subjects classified as meningitis cases by laboratory test.

From May 2022 to February 2023, the Ministry of Health detected 1801 subjects exposed to neuraxial blockade in 4 private hospitals in Durango, Mexico. Case definitions were established for the active subject search and classified into 4 groups. Exposed cases were those subjects who had undergone neuraxial blockade at these hospitals during this period. Suspected cases were all exposed subjects with symptoms suggestive of meningitis (headache, stiff or painful neck, seizures, mental alterations, nausea, vomiting) while the probable cases were all exposed or suspected cases with the presence of at least 1 CSF alteration (hypoglycorrhachia, hyperproteinorrachia, and pleocytosis). Finally, meningitis cases were classified according to 2 criteria: epidemiological-clinical association (symptoms and/or CSF alterations not explained by other causes) or laboratory test confirmation (culture or qPCR for *F solani*) (Table 1). The case search was conducted through telephone calls and home visits to reach exposed subjects for referral to public health hospitals in Durango for clinical evaluation. To illustrate the search for meningitis cases, Figure 1 shows the procedures followed to identify, classify, and treat subjects during the outbreak. Exposed cases with severe symptoms were hospitalized, underwent lumbar puncture, and then classified as meningitis cases and treated with antifungal drugs after isolation of *F solani*.

Exposed cases with mild symptoms were evaluated and a lumbar puncture was performed. Patients with CSF alteration were classified as meningitis cases, hospitalized, and treated. However, 5 cases without CSF alteration and negative qPCR test, but with persistent symptoms, were classified as meningitis cases, admitted to the hospital, and received antifungal drugs. Additionally, after the screening of 838 subjects, 3 exposed cases without symptomatology that showed CSF alterations were identified and classified as meningitis cases, while the rest of the exposed cases were followed up in an ambulatory setting (Figure 1)

**Figure 2.**
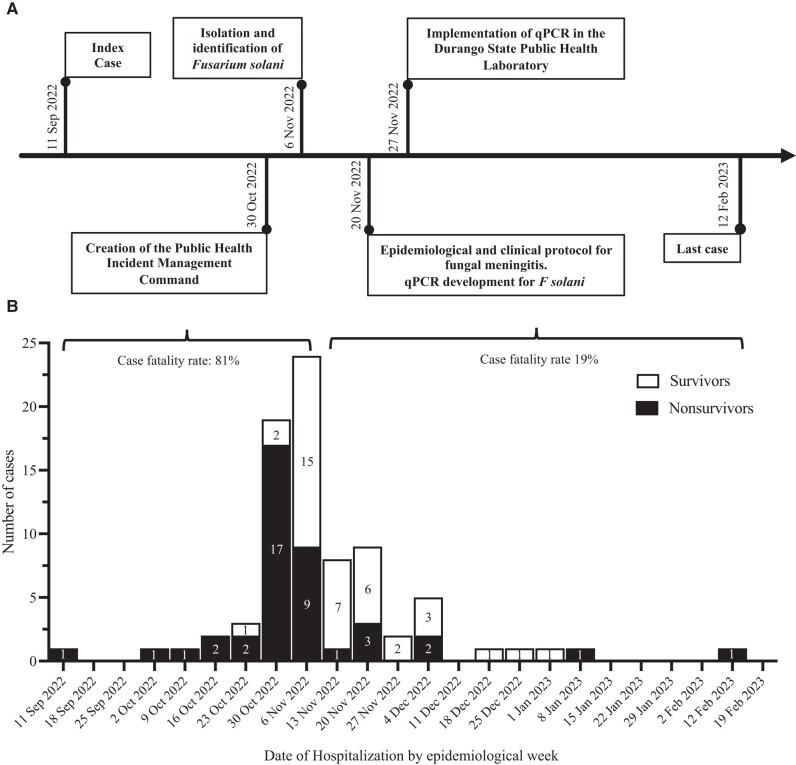
Epidemiological history of the Durango outbreak. *A*, Timeline of healthcare actions implemented by the Public Health Incident Management Command during the outbreak. *B*, Number of hospitalized cases and clinical outcomes: nonsurvivors (black bar) or survivors (white bar), according to epidemiological week. The numbers within the bar indicate the number of cases. Abbreviation: qPCR, real-time polymerase chain reaction test.

The number of meningitis cases by date of hospitalization and the clinical outcome of those patients is shown in Figure 2, where the first 5 cases had an unfavorable outcome. It is observed that the last week of October and the first week of November registered the highest number of hospitalizations with 19 and 24, respectively, of which 26 patients were nonsurvivors (81% of the total deaths) and 17 survivors. In addition, in these 2 weeks, Durango's hospitals began the implementation of the health policies of the Public Health Incident Management Command. From 11 November, a reduction in hospitalizations and a lower proportion of deaths (19% of the total) was observed (Figure 2).

### Identification of *Fusarium solani* in Cases of Meningitis

During the outbreak, it was not possible to isolate etiological agents from CSF until 11 November, when brain biopsies of 2 patients were processed at INCMNZS for identification and fungal susceptibility testing. The blood agar plates had filamentous fungi growth, and a blue lactophenol stain revealed septated hyphae with curved banana-shaped macroconidia characteristic of *Fusarium* sp. Mass spectrometry analyses with scores >2 and Sanger sequencing of the EF1α gene with 100% coverage revealed the presence of the *Fusarium solani* sp complex. Antifungal susceptibility testing by broth microdilution was also performed on 3 samples to determine the minimum inhibitory concentration (MIC) of 4 antifungal drugs used for fusariosis [11]. Susceptibility testing of 3 clinical isolates indicated that voriconazole and AmB had the lowest MIC with 2 μg/mL and 2–4 μg/mL, respectively, compared to itraconazole and posaconazole (>16 μg/mL), which allowed the choice of the antifungal medication.

### Retrospective Analysis

Among 838 subjects screened by CSF alteration and qPCR, 80 subjects were classified as meningitis cases; 31 cases were confirmed by laboratory test and 49 by clinical-epidemiological association (Table 2), with a case fatality rate of 51.3% (41 patients) and a median age of 30 years (interquartile range [IQR], 24.7–36.2 years) with no difference between those who died and those who survived. Most patients were females (95%) who had received a variety of surgical procedures, with c-section being the most common (76.3%). A total of 57 patients (72.6%) were screened by qPCR; 31 subjects had a positive result and 26 were negative, representing 38.8% and 32.5%, respectively. Furthermore, in 23 patients (28.7%), the qPCR test for *F solani* was not performed (Table 2). Of 41 who died, 19 patients (46.3%) had a positive qPCR result, and 2 patients (4.9%) were qPCR negative, while in the case of survivors, 12 patients (30.8%) had a positive result and 24 patients (61.5%) had a negative one. The median incubation period, the time between surgery and symptom onset, was 7 days (IQR, 4–30 days), being similar in nonsurvivors (7 days [IQR, 4–30.5]) and survivors (7 days [IQR, 3.7–25.7]). In overall, the median length of hospitalization was 39.5 days (IQR, 24.7–36.2 days), whereas in nonsurvivors was 50.5 days (IQR, 18.7–84.5 days), and in survivors 38 days (IQR, 18–93 days), although this difference was not significant. In addition, the time since surgery to hospital admission was similar as observed in all patients (78 days [IQR, 52.5–99.7 days]), those who died (76 days [IQR, 55.5–93.5] days), and survivors (80 days [IQR, 38–126]). Additionally, 45% of patients (36 patients) were admitted to ICU, 34 patients (82.9%) had a fatal outcome, and 2 patients (5.1%) showed clinical improvement (*P* < .001), while the rest died in other hospital areas such as emergency room or intermediate therapy (Table 2).

**Table 2.** Demographic and Clinical Data From Patients With Meningitis Associated With *Fusarium solani* Infection (N = 80)

Characteristic	Total	Nonsurvivors	Survivors	*P* Value
Meningitis cases, No. (%)	80 (100)	41 (51.3)	39 (48.7)	
Age, y, median (IQR)	30 (24.7–36.2)	30 (22.5–35)	30 (25–37.2)	.942^a^
Female, No. (%)	76 (95)	40 (97.6)	36 (92.3)	.353^b^
Surgical procedures, No. (%)				
Cesarean section	61 (76.3)	33 (80.5)	28 (71.8)	.258^b^
Cholecystectomy	3 (5.1)	2 (4.9)	1 (2.6)	
Knee arthroplasty	3 (3.8)	2 (4.9)	1 (2.6)	
Hysterectomy	2 (2.5)	0 (0)	2 (5.1)	
Ovarian cystectomy	2 (2.5)	2 (4.9)	0 (0)	
Natural childbirth	2 (2.5)	0 (0)	2 (5.1)	
Orchiectomy	2 (2.5)	0 (0)	2 (5.1)	
Bilateral tubal occlusion	1 (1.3)	0 (0)	1 (2.6)	
Colpoperineoplasty	1 (1.3)	0 (0)	1 (2.6)	
Hysteroscopy	1 (1.3)	0 (0)	1 (2.6)	
Osteosynthesis removal	1 (1.3)	1 (2.4)	0 (0)	
qPCR results, No. (%)				
Positive	31 (38.8)	19 (46.3)	12 (30.8)	**<.001** ^b^
Negative	26 (32.5)	2 (4.9)	24 (61.5)	
Without qPCR	23 (28.7)	20 (48.8)	3 (7.7)	
Time periods, median (IQR)				
Incubation days^c^	7 (4–30)	7 (4–30.5)	7 (3.7–25.7)	.836^a^
Days of hospitalization^d^	39.5 (18–86.5)	50.5 (18.7–84.5)	38 (18–93)	.338^a^
Days since surgery to hospitalization	78 (52.5–99.7)	76 (55.5–93.5)	80 (38–126)	.149^a^
Antifungal onset^e^, days	4 (2–9)	7 (3–11.7)	3.5 (2–6.2)	.**018**^a^
Patient management, No. (%)				
Admission to the ICU	36 (45)	34 (82.9)	2 (5.1)	**<**.**001**^b^
Treatment with voriconazole and AmB	71 (88.8)	35 (85.4)	36 (92.3)	.483^b^
Treatment with steroids	64 (80)	35 (85.4)	29 (74.3)	.122^b^

Abbreviations: AmB, amphotericin B; ICU, intensive care unit, IQR, interquartile range, qPCR, real-time polymerase chain reaction. Bold values indicate a significant difference.

^a^Pearson χ^2^.

^b^Kruskal-Wallis.

^c^Incubation days: Days elapsed between surgical procedure and symptom onset.

^d^Days of hospitalization: Time from hospital admission until discharge or death.

^e^Voriconazole and AmB onset time: Time from hospitalization until administration of antifungal treatment.

Voriconazole and AmB were used in 71 patients (88.8%), with a similar number of nonsurvivors (35 patients) and survivors (36 patients), while 9 patients died before medication was started (*P* = .483). In addition, it was observed that patients who had a fatal outcome received antifungal drugs 7 days (IQR, 3–11.7 days) after hospitalization, whereas survivors were treated in 3.5 days (IQR, 2–6.2 days) (*P* = .018). Additionally, 64 patients (80% of the total) received steroids during their hospitalization; of them, 35 patients (85.4%) had a fatal outcome, and 29 patients (74.3%) were survivors (*P* = .122).

**Figure 3.**
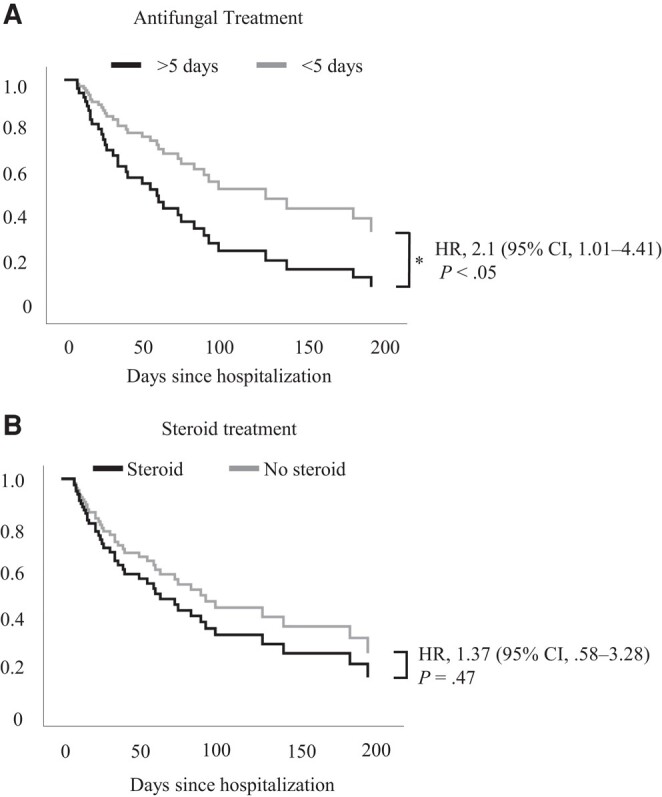
Opportune treatment is associated with better survival outcomes. Cox proportional hazard regression analysis for patients who received antifungal treatment before and after 5 d since hospitalization date (*A*) or patients with and without steroid administration during hospital stay (*B*). *P < .05. Abbreviations: CI, confidence interval; HR, hazard ratio.

To evaluate the association of Public Health Incident Management Command actions on healthcare improvement, the association of time onset of antifungal drugs and steroid use in clinical outcomes was determined. In this outbreak, the knowledge about the etiological agent allowed modifying the administration time of voriconazole and AmB after hospitalization (55 days for the index case compared to 1 day for the last case). In-depth analysis of antifungal treatment start, through the ROC curve, showed an AUC of 0.696, while the Youden index revealed a therapeutic time window of voriconazole/AmB of <5 days from hospital admission, with a sensitivity of 0.72 and specificity of 0.31 (*P* < .05) (Supplementary Figure 1). This therapeutic time window was associated with better survival outcomes, considering that late voriconazole/AmB administration (after 5 days) confers a lethality risk of 2.1 (95% confidence interval [CI], 1.01–4.48, *P* < .05), according to Cox proportional hazard regression (Figure 3*A*). In contrast, the use of steroids showed no differences between deaths and survivors (Table 2) and steroid administration was not associated with better or worse outcomes, as observed in the Cox regression curve (HR, 1.37 [95% CI, .58–3.28], *P* = .47) (Figure 3*B*). Together these results show that despite the high mortality rate of this outbreak, the actions taken—such as fungi identification, timely treatment, and healthcare management—helped to respond to this challenging scenario.

## DISCUSSION

The Durango emergency represented the largest outbreak of meningitis associated with *F solani* infection and the third related to surgical procedures with epidural anesthesia in the world [6, 7]. Fusariosis in CNS is rare in humans; only 26 cases were reported between 1974 and 2022, with an overall lethality of 72%. Most of these patients suffered from hematological malignancies or immunosuppressive conditions, both of which are risk factors for the development of invasive fusariosis [12]; only 1 case of intracranial fusariosis in an immunocompetent subject has been reported [13]. This previous evidence along with the high lethality rate (51.3%) observed in the Durango outbreak supports the notion that this is a life-threatening disease. Furthermore, observing 2 similar outbreaks in a very short time highlights the need to expand the current knowledge about meningeal fusariosis. A retrospective study from 11 countries showed that 233 immunocompromised subjects developed invasive fusariosis in the skin, lungs, or blood; however, none of them presented neurological disease [14]. Meningeal fusariosis was demonstrated in immunodeficient mice, where direct administration of this fungus into the bloodstream is sufficient to induce systemic fusariosis, reflecting the potential invasiveness of this phytopathogen [15] and suggesting that the optimal function of the immune system is crucial to prevent invasive fusariosis. The data exhibited here disclosed the case series of fungal meningitis due to *Fusarium solani* infection in immunocompetent patients, unveiling its capabilities of infecting the CNS even in persons without risk factors.

Evidence from mice models showed that administration of high-inoculum doses of *Fusarium solani* was associated with high mortality [15], which could explain the high case fatality rate and the different outcomes observed in this outbreak. For instance, qPCR-negative patients with symptoms and CSF alterations responded to antifungal treatment, suggesting that they probably received low fungi doses that were not detectable in the CSF. A previous study shows that the yield of PCR for the diagnosis of invasive fungal infections depends on the biopsy type and sample amount [16], which may be a limitation of molecular testing in meningeal fusariosis. This emergency raises awareness about potential etiological microorganisms in cases of atypical meningitis and highlights the need to explore the pathophysiology of meningeal fusariosis for a comprehensive understanding of the disease.

Durango's outbreak represented an enormous challenge for the Mexican health sector that required the coordination of the Public Health Incident Management Command, collaboration between local and federal entities, and experience-sharing with international agencies, such as the US Centers for Disease Control and Prevention, to improve patient healthcare. Most deaths (33 patients) occurred in patients hospitalized before 6 November, when medical staff had less knowledge about clinical management for this atypical meningitis. However, the actions taken by the health sector were crucial to the outbreak response. For instance, the identification of *F solani* was essential to know that meningitis was associated with fungi and to address the isolation protocol and antifungal susceptibility testing. These actions determined that a combination of voriconazole and AmB could be effective against the infection, allowing physicians to initiate antifungal treatment in some patients, even before qPCR test implementation. Other important actions during the outbreak were the development of the clinical protocol for the medical care of patients and qPCR implementation as a diagnostic test. The protocol served as a guide for physicians, providing knowledge about meningeal fusariosis, warning about signs and symptoms, and medication management. One relevant aspect of medical care was the use of steroids, which are indicated for bacterial meningitis showing a positive impact on survival [17]. Nonetheless, the data obtained from Durango's outbreak indicate that steroid administration was not associated with good or poor clinical outcomes, although it was useful in reducing symptomatology. On the other hand, qPCR implementation provided an accurate diagnosis in less time (compared to culture isolation), allowing earlier initiation of antifungal treatment, demonstrating that qPCR is a feasible technology for diagnosis and potential use in future fungal outbreaks. The proper use of antifungal medications within 5 days of hospital admission is associated with better clinical outcomes and higher overall survival rates; however, epidemiological, medical, and diagnostic actions may also have contributed to patient survival.

The Mexican Ministry of Health established the ending of the Durango outbreak by the time the equivalent of 2 long incubation periods of 4.8 months (9.6 months) had elapsed, from the last reported case in February 2023. During the current year, a second binational outbreak of meningitis associated with epidural anesthesia was reported on the Mexico–US border, with a 3-month difference between the last case in Durango and the first in Matamoros, Tamaulipas [7]. There is valuable information on these similar outbreaks that could be important to analyze.

Mexican and US authorities established similar definitions for exposed and suspected cases, where the exposition to neuraxial blockade and symptoms that suggest CNS infection were considered. In the Durango outbreak, probable cases were identified by CSF alterations such as hypoglycorrhachia, hyperproteinorrachia, or pleocytosis, whereas in Matamoros, a white blood cell count >5 cells/mm^3^ was the parameter considered. These evaluations set up a guideline for physicians; however, the diagnostic accuracy of these determinations or even the pertinence to include other analytes such as β-D-glucan in the meningeal fusariosis context remains unknown.

Regarding meningitis cases in Durango, the clinical-epidemiological association was fundamental to identify these cases, especially before qPCR implementation or in patients with persistent symptoms and CSF alterations. In the Matamoros emergency, meningitis cases were only classified by molecular tests such as PCR and metagenomic sequencing. Another challenging aspect during the outbreaks was to determine the appropriate drug treatment. In Durango, MIC testing revealed that a combination of voriconazole/AmB was suitable for patient treatment. In contrast, the isolated strain from the Matamoros outbreak showed a wide antifungal resistance, leading to exploring novel antifungal treatments such as fosmanogepix, in combination with voriconazole and AmB.

Despite all implemented actions and the enormous effort made to respond to outbreaks in Mexico and the US, there was a fatality rate of 51.3% for the Durango outbreak and 80% for the Matamoros one, demonstrating that this challenging health issue requires further research to understand the pathophysiology of the disease, improve diagnostic tests, and develop new treatment approaches to provide optimal care to patients.

Finally, the work presented here exhibits the elevated health risk of meningeal fusariosis; the actions taken to respond to this scenario, such as a practical diagnosis by molecular testing; and the opportunity for targeted treatment and clinical management. The data exposed could be supporting evidence for managing the ongoing binational outbreak in Matamoros or another outbreak related to *F solani*.

## CONCLUSIONS

This outbreak highlights the relevance of pathogenic fungi in neurological diseases that can become public health issues. The clinical awareness obtained during the outbreak, the identification of the etiological agent, and the timely initiation of treatment resulted in better outcomes. Finally, our experience during this outbreak underscores the importance of coordinating public health actions to reduce suffering, manage risks, and prevent emerging crises.

## Supplementary Material

ofad690_Supplementary_Data

## Supplementary Data

Supplementary materials are available at *Open Forum Infectious Diseases* online. Consisting of data provided by the authors to benefit the reader, the posted materials are not copyedited and are the sole responsibility of the authors, so questions or comments should be addressed to the corresponding author.

## Notes

***Author contributions.*** G. G.-R., I. K.-P., C. A. Z.-J., V. B. G.-C., R. F.-A., T. V.-R., and A. V.-R. gathered, analyzed, and updated epidemiological information. C. D.-M., I. K.-P., L. A. R.-C., J. C. T.-F., and H. C. S.-S. recorded and validated patients’ demographic and clinical data. R. A. M.-G., and F. G.-L. were involved in isolating and identifying *Fusarium solani*. I. L. M. developed and standardized the qPCR test. L. C. B., R. R.-R., and O. C.-E. analyzed and interpreted demographic and clinical data. T. M. C. contributed intellectually and reviewed the manuscript. O. C.-E., L. C.-B., R. R.-R., R. P., G. G.-R., and C. D.-M. wrote the manuscript. G. C.-R., C. D.-M., Z. R.-A., and J. A.-V. conceived and designed the study.

***Acknowledgments*.** We acknowledge all professional medical staff involved in the strategy implementation and response to this challenging outbreak, particularly, Dr. Ramiro López-Elizalde, Dr. Michelle Herrera-Canales and Dr. Marcela Pelayo-Nieto from the Institute for Social Security and Services for State Workers, as well as Dr. Dinael Beltrán-Santiago, Dr. Nancy Esmeralda Castro-Veloz, Dr. Christian Alan García-Estrada, Dr. Miguel David Castruita-Flores and Dr. Héctor Patiño-Rubio from the Mexican Institute of Social Security.

***Patient consent*.** This study does not include factors necessitating patient consent.

***Disclaimer*.** The findings and conclusions of this work do not represent the official position of the Mexican health institutions.

***Financial support*.** The Mexican Ministry of Health supported the strategy implementation and this work.
