# Supplementary material for: Outbreak of Fusarium solani Meningitis in Immunocompetent Persons Associated With Neuraxial Blockade in Durango, Mexico, 2022–2023
Source: Open Forum Infect Dis. 2024 Jan 4;11(2):ofad690. doi: 10.1093/ofid/ofad690 (PMC10873708; doi:10.1093/ofid/ofad690)
Supplement: ofad690_Supplementary_Data [file ofad690_supplementary_data.pdf]

ROC of Antifungal Treatment

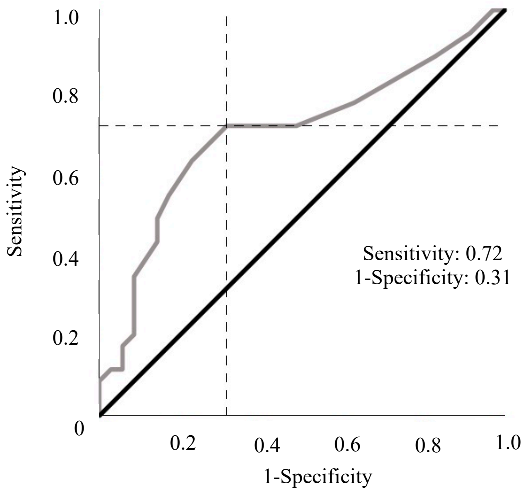

Area Under Curve	SE	p-value	95% Confidence Interval	
			Lower limit	Upper limit
0.696	0.065	0.005	0.569	0.823

Supplementary figure 1. Voriconazole and Amphotericin B therapeutic time window. The Receiver Operator Characteristic (ROC) curve of antifungal treatment onset days during hospitalization stays and its association with death and survival outcomes. Abbreviations: CI, Confidence interval.
